# Three Component Reaction: An Efficient Synthesis and Reactions of 3,4-Dihydropyrimidin-2(1*H*)-Ones and Thiones Using New Natural Catalyst

**DOI:** 10.1155/2013/706437

**Published:** 2013-08-18

**Authors:** A. M. Elmaghraby, I. A. Mousa, A. A. Harb, M. Y. Mahgoub

**Affiliations:** Department of Chemistry, Faculty of Science, South Valley University, Qena 83523, Egypt

## Abstract

Synthesis of 3,4-dihydropyrimidin-2(1*H*)-one and 3,4-dihydropyrimidin-2(1*H*)-thione derivatives from aldehydes, 1,3-dicarbonyl derivatives and urea or thiourea using granite and quartz as new, natural and reusable catalysts. Some of the 3,4-dihydropyrimidin-2(1*H*)-thione derivatives were used to prepare new heterocyclic compounds. The antimicrobial activity of selected examples of the synthesized compounds was tested and showed moderate activity.

## 1. Introduction

Aryl-3,4-dihydropyrimidines derivatives have recently received great attention because of their wide range of therapeutic and pharmacological properties, such as antiviral [1], antitumor, antibacterial and antifungal [2], anti-inflammatory [3], antihypertensive agents, and neuropeptide Y (NPY) antagonists [4]. Furthermore, these compounds have emerged as the integral backbones of several calcium-channel blockers [5]. Also, several alkaloids containing the dihydropyrimidine were isolated from marine sources, for example, of these are the batzelladine alkaloids, which are found to be potent HIVgp-120-CD4 inhibitors [6, 7].

In general, the classic Biginelli approach to 3,4-dihydropyrimidinones is based on the condensation of ethyl acetoacetate, aromatic aldehyde, and urea under strong acidic conditions; this suffers, however, from low yields of products, particularly in case of substituted aromatic and aliphatic aldehydes [8, 9]. This problem has led to the development of multistep synthetic strategies that produce relatively higher yields, but lack the simplicity of the original one-pot-Biginelli protocol. Thus, the Biginelli reaction has received renewed interest from researchers interested in discovering milder and more efficient procedures that are applicable to a wide range of substituents in all three components and proceed in better yields. So, the one-pot-Biginelli protocol for 3,4-dihydropyrimidines synthesis was explored by varying all components and catalysts [10–18] in protic, aprotic solvents, and solvent free conditions [19] using either classical heating, microwave [20, 21], ultrasound [22, 23], and visible light (100 W Lamp, THF) irradiations [24]. Also several improved procedures have been reported recently using not only acidic media such as Lewis acids, protic acids, and ionic liquids as promoters [25, 26] but also nonacidic substances such as baker's yeast [27], graphite [28], and iodine [29, 30]. Heterogeneous solid acids are used also; however, these are advantageous over conventional homogeneous acid catalysts as they can be easily recovered from the reaction mixture by simple filtration and can be reused after activation or without activation, thereby making the process economically viable [31]. Bakibaev and Filimonov [32] reported that piperidine as a base catalyst can promote the Biginelli protocol also, to afford the corresponding 3,4-dihydropyrimidines along with Hantzsch 1,4-dihydropyridines which may form in spite of urea decomposition in the reaction media, releasing ammonia. We would like to propose a new naturally and very cheap catalysts granite and quartz for the synthesis of 3,4-dihydropyrimidinones and 3,4-dihydropyrimidenthiones, using one-pot-Biginelli protocol, in refluxing ethanol. 

## 2. Result and Discussion

**Scheme 1:**
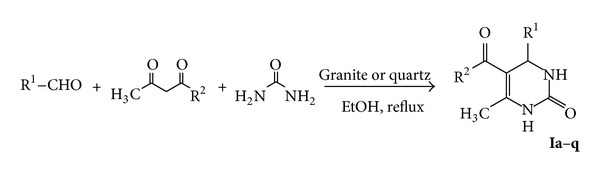


It is interesting to report that the one pot reaction of a mixture of benzaldehyde, ethyl acetoacetate, and urea in the presence of granite or quartz as a catalyst in refluxing ethanol resulted in the formation of 4-phenyl-3,4-dihydropyrimidinone **Ia**, Table 1 in 64% or 68% yield according to the catalyst (Scheme 1). In a similar way, urea was condensed smoothly with variety of aromatic or heterocyclic aldehydes and variety of 1,3-dicarbonyl compounds in the presence of granite or quartz in refluxing ethanol as one pot reaction to afford the corresponding 3,4-dihydropyrimidines **Ib**–**q** (Table 1) whose composition and structures were confirmed by elemental analysis, Mass, IR, and ^1^H NMR spectra of the isolated products (*cf*. experimental section). 

**Scheme 2:**
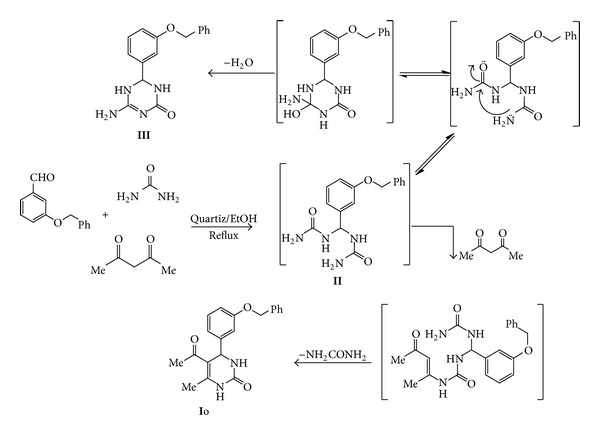


On the other hand, carrying out of the above reaction using of 3-benzyloxybenzaldehyde, acetyl acetone, and urea in refluxing ethanol using granite as catalyst, the corresponding 5-acetyl-4-(3-(benzyloxy)phenyl)-6-methyl-3,4-dihydropyrimidin-2(1*H*)-one **Io** was isolated. However, on carrying the above reaction using quartz as a catalyst, beside the proposed 3,4-dihydropyrimidinone **Io**, another product with molecular formula (C_16_H_16_N_4_O_2_), *m*/*z* = 296 was isolated from the reaction media in 25% yield. This product can be identified as 4-amino-6-(3-(benzyloxy)phenyl)-5,6-dihydro-1,3,5-triazin-2(1*H*)-one **III** based on the analytical and the spectral data of the isolated product, which revealed the presence of characteristic stretching vibrations due to NH, NH_2_, and amidic CO at *ν* = 3450, 3300, and 1640 cm^−1^ regions, respectively, in the IR spectrum. Also, the ^1^H-NMR spectrum of the isolated product shows signals at *δ* = 5.07 (s, 2H, –CH_2_–), 5.43 (s, 1H, –CH–), 5.68 (s, 2H, –NH_2_), 6.70–7.46 (m, 9H, Ar), and 10.0 (s, 1H, NH) ppm. The ^13^C-NMR spectrum of the isolated product shows signals at *δ* = 51.2 (CH aliphatic), 62.32 (CH_2_ aliphatic), 165.3 (C triazine ring), 190.4 (C=O amidic), and 111–160 (Benzene rings). This expectation is based on the observation that the 3-benzyloxybenzaldehyde condensed with two moles of urea to give the corresponding bis-ureide **II** as key intermediate which cyclized *via* elimination of H_2_O to give the extremely low yield triazine derivative **III** [44] (Scheme 2).

**Scheme 3:**
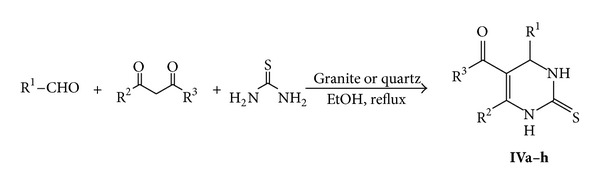


 In generality of this process, various 1,3-diketones and aldehydes were reacted with thiourea in refluxing ethanol using granite or quartz as the reaction catalyst to give the corresponding 3,4-dihydropyrimidin-2(1*H*)-thione derivatives **IV** (Table 2) which their structures were confirmed on the bases of the analytical and spectral data of the isolated products (*cf*. experimental section) (Scheme 3).

On reading of the experimental results, we noted that aromatic aldehydes carrying either electron-donating or electron-withdrawing substituents reacted well under the reaction conditions to give the corresponding products in moderate to good yields high purity in case of granite or quartz. However, the obtained yields on using quartz are higher than granite either in case of urea or thiourea. This may be due to the high percentage of SiO_2_ in quartz. This procedure not only preserves the simplicity of the Biginelli reaction but also produces good yields of the products with high purity. Also, the catalyst was recovered by simple filtration and reused in subsequent reactions with consistent activity.

We can use the prepared 3,4-dihydropyrimidenthiones **IVa**–**c** to synthesize newly derivatives. Thus, heating of **IVa–c **with methyl iodide in dry acetone in the presence of anhydrous potassium carbonate afforded the S–CH_3_ derivatives **Va**–**c** which was confirmed by using elemental analysis and spectral data. The ^1^H NMR illustrated the presence of singlet S–CH_3_ protons.

On the other hand, heating of **IVa**–**c **in acetic anhydride afforded the corresponding 3-*N*-acetyl derivatives **VIa**–**c**. Structure **VIc** was deduced from elemental and spectral data. The mass spectrum showed the molecular ion peak at *m*/*z* (%) = 378 (M^+^, 48.03), for molecular formula C_18_H_22_N_2_O_5_S. The ^1^H NMR illustrated the presence singlet N–COCH_3_ protons at *δ* = 2.60 ppm, in addition to other singlet peaks at *δ* = 2.27, 3.67, and 3.77 ppm for methyl and two methoxy groups, respectively, and the absence of the NH proton at *δ* = 7.27. 

**Scheme 4:**
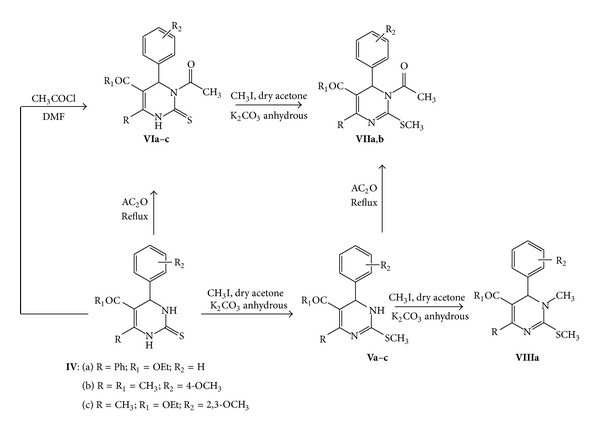


In the same time, we can use the same conditions to prepare **VIa,b** which was elucidated by correct elemental analysis and spectral data (*cf.* experimental data). Also **VIa**–**c** was synthesized by the reaction of **IVa**–**c** with acetyl chloride in DMF (melting and mixed melting point) (Scheme 4).

In the same time, the pyrimidine derivatives **VIIa,b** can be synthesized via acetylation of the corresponding S–CH_3_ derivatives **Va,b** using acetic anhydride. Also, it can be prepared via methylation of the *N*-acetyl derivatives **VIa,b**. Structures **VIIa,b** were elucidated by elemental analysis and spectral data. The mass spectrum for **VIIa** showed the molecular ion peak at *m/z* (%) = 394 (M^+^, 12.51), while **VIIb** illustrated the molecular ion peak at *m*/*z* (%) = 332 (M^+^, 43.22). The ^1^H NMR revealed the presence of singlet peak at *δ* = 2.50 ppm for COCH_3_ protons and the absence of the singlet peak at *δ* = 7.27 ppm for NH proton. Also IR spectrum showed the absence of NH peak (Scheme 4).

Methylation of **Va** was carried out in methyl iodide in DMF in the presence of K_2_CO_3_ anhydrous that yielded **VIIIa** which was confirmed by correct elemental analysis as well as spectral data. The ^1^H NMR showed the absence of singlet peak at *δ* = 7.27 ppm for NH proton and the appearance of a singlet peak at *δ* = 3.33 ppm for N–CH_3_ protons (Scheme 4).

**Scheme 5:**
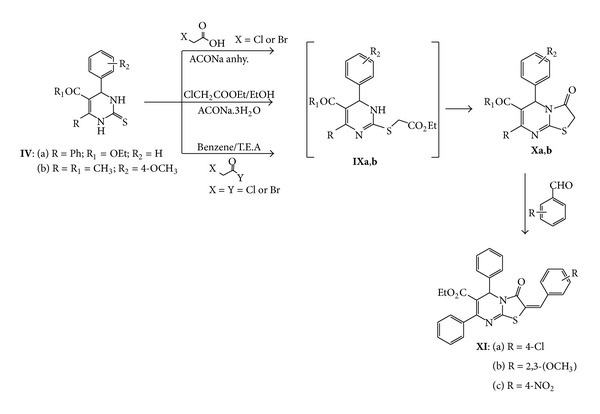


Heating of **IVa** with ethylchloroacetate in ethanol and sodium acetate afforded ethyl 3-oxo-5,7-diphenyl-3,5,8,8a-tetrahydro-2*H*-thiazolo[3,2-*a*]pyrimidine-6-carboxylate **Xa **over the unisolated intermediate ethyl 2-(2-ethoxy-2-oxoethylthio)-4,6-diphenyl-1,6 dihydropyrimidine-5-carboxylate **IXa** as shown in elemental analysis as well as spectral data. The mass spectrum showed the molecular ion peak at *m/z* (%) = 378 (M^+^, 60.03) for molecular formula C_21_H_18_N_2_O_3_S. The ^1^H NMR revealed also the presence of one only ethyl ester group, at *δ* = 0.85 for CH_3_ protons (t) and 3.85 for CH_2_ (q), and also the absence of NH proton at *δ* = 7.27 ppm. The IR spectrum showed absorption bands at 1752, 1675, and 1589 cm^−1^ for carbonyl ester, amidic carbonyl groups, and C=N, respectively. Also, the isolated product **Xa** was obtained *via* the reaction of **IVa** with chloroacetyl chloride or bromoacetyl bromide in benzene and drops of triethylamine as catalyst. In the same time, compound **Xa** can be isolated from the reaction of **IVa** with chloro- or bromoacetic acid in acetic acid and acetic anhydride mixture in presence of anhydrous sodium acetate. Similarly, compound **Xb **was prepared from the reaction of **IVb** with ethylchloroacetate, chloroacetic acid, or chloroacetyl-chloride as shown in previous conditions (Scheme 5). 

Compound **Xa** was condensed with different aromatic aldehydes in refluxing ethanolic pipredine solution to give the corresponding arylidene derivatives **XIa**–**c**. Structures **XIa**–**c **were deduced from its elemental analysis and spectral data. The ^1^H NMR showed the absence of singlet peak for CH_2_ protons at *δ* = 3.88 ppm and the appearance of singlet peak for =CH proton at *δ* = 7.74 ppm (Scheme 5).

**Scheme 6:**
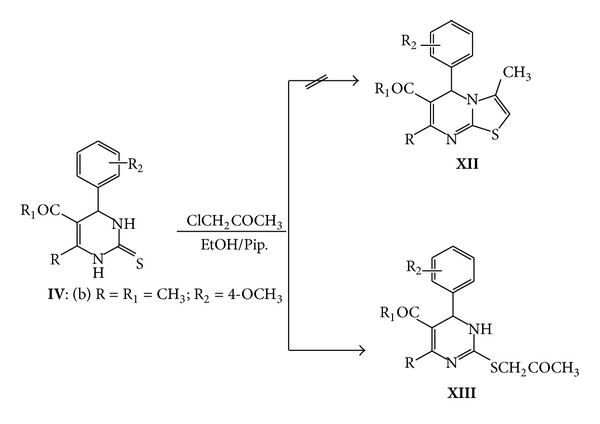


Aiming to the synthesizing of thiazolopyrimidine **XII,** we refluxed **IVb** with chloroacetone in ethanolic piperidine solution. However the corresponding 1-(5-acetyl-6-(4-methoxyphenyl)-4-methyl-1,6-dihydropyrimidin-2-ylthio)propan-2-one **XIII **was formed which was identified by elemental analysis as well as spectral data. The mass spectrum showed the molecular ion peak at *m*/*z* (%) = 332 (M^+^, 5.30) for molecular formula C_17_H_20_N_2_O_3_S. The ^1^H NMR confirmed the presence of only one NH proton at *δ* = 7.11 ppm and singlet peak at *δ* = 2.46 ppm due to CH_2_ protons (Scheme 6).

**Scheme 7:**
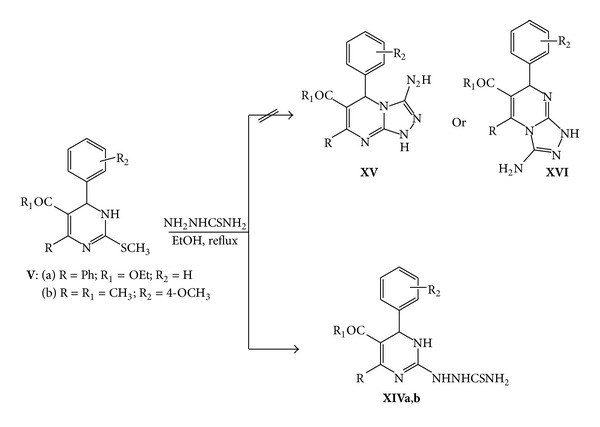


On the other hand, compound **Va,b** was reacted with thiosemicarbazide in refluxing ethanol to give the corresponding carbazide **XIVa,b** instead of the corresponding fused pyrimidinotriazoles **XV** and **XVI**. Structures **XIVa,b** were established by elemental analysis and spectral data where the mass spectrum showed the molecular ion peak at *m*/*z* (%) = 395 (M^+^, 24.13) for **XIVa** and at *m/z* (%) = 333 (M^+^, 12.18) for **XIVb** (Scheme 7).

**Scheme 8:**
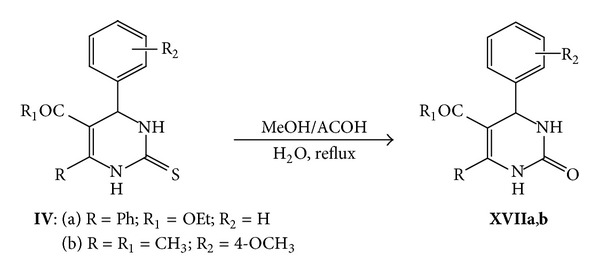


On the other hand, refluxing of **IVa,b **in methyl alcohol in the presence of acetic acid and water (4 : 1 : 1) afforded 3,4-dihydropyrimidinone derivatives **XVIIa,b**. Compound **XVIIa** was confirmed by compare mp. (Found) = 160°C, mp. (Reported) = 158°C [48]. Also, compound **XVIIb** was established by compare mp. (Found) = 164–166°C; mp. (Reported) = 166-167°C [48] (Scheme 8).

## 3. Antimicrobial Activity

There are 5 compounds (**III**,** IVg**,** IVf**,** IVh**, and** IVc**) that were tested and showed promising positive antibacterial activity.

All the compounds showed activity against bacteria such as *Staphylococcus aureus *and* Escherichia coli.* The **IVh** & **IVc** compounds showed positive antibacterial against *S. aureus *which are 14.5 mm and 14 mm, respectively, which are 0.25 and 0.75 mm less than the zone around Streptomphenicol disc. This may be due the presence of sulfur atomand pyrimidine ring.

The other three most active compounds tested are compounds **IVg**, **IVf**, and **III**. The activity of these compounds against *Staphylococcus aureus *showed positive reactions, 12.75, 12.5, and 12 mm of inhibition zones, respectively, compared to the inhibition zone of antibiotic used, as indicated in (Table 3); this may be due to sulfur atom, two chlorine atoms, and triazine ring, respectively.

All the compounds have approximately the same effect against *Escherichia coli* bacteria as indicated by the zone of inhibition (Table 3). In case of using these compounds as antimicrobial cytotoxicity, effect of these compounds must be examined.

## 4. Conclusion

In summary, we have found that quartz and granite are extremely useful and highly efficient new natural, solids for the synthesis of biologically potent aryl 3,4-dihydropyrimidines by means of three-component condensations of an aldehyde, 1,3-dicarbonyl compound, and urea or thiourea in a one-pot operation. This method is applicable to a wide range of substrates, including aromatic and heterocyclic aldehydes, and provides a variety of biologically relevant 3,4-dihydropyrimidinones and 3,4-dihydropyrimidinthiones in high yields after short reaction times. 

## 5. Experimental Section

### 5.1. General

All melting points were measured with a Gallenkamp apparatus. The IR spectra of samples were recorded in KBr via a Shimadzu FT-IR 8101 PC infrared spectrophotometer. ^1^H NMR spectra were run at 300 MHz and recorded in CDCl_3_/[D6] DMSO using TMS as the internal standard. Chemical shifts were related to that of the solvent. Mass spectra were measured on a GCMS-QP1000 EX spectrometer at 70 eV. TLC was conducted on 0.25 mm precoated silica gel plates (60F-254). Elemental analyses were carried out at the Microanalytical Center of Cairo University, Giza, Egypt. The catalyst is ground until it became fine powder.

#### 5.1.1. General Procedure for the Synthesis of the Newly 3,4-Dihydropyrimidinones **(Io,p,q)** and 3,4-Dihydropyrimidinthiones **(IVc,h,g,f)**

A mixture of aldehyde (1 mmol), 1,3-dicarbonyl compounds (1 mmol), urea or thiourea (1 mmol), and granite or quartz (0.5 g) in ethanol (15 mL) was heated under reflux for the required time. After completion of the reaction as monitored by T.L.C., the reaction mixture was filtered to separate the catalyst. Keep the reaction mixture overnight. The solid product was filtered under suction then recrystallized from ethanol to afford pure product. 

*Ethyl-4-(3-(benzyloxy)phenyl)-6-methyl-2-oxo-1,2,3,4-tetrahydropyrimidine-5-carboxylate* **(Io)**. mp. = 178–180°C. I.R (KBr): *ν* = 3300, 3100, 2950, 1700, 1630 cm^−1^. ^1^H NMR (300 MHz, CDCl_3_): *δ* = 1.16 (t, *J* = 7.2 Hz, 3H, –O–CH_2_–CH_3_), 2.33 (s, 3H, CH_3_), 4.08 (q, 2H, –O–CH_2_–CH_3_), 5.03 (s, 2H, –O–CH_2_–Ph), 5.37 (s, 1H, –CH–), 5.92 (s, 1H, –NH), 6.85–6.94 (m, 4H, Ar), 7.19–7.39 (m, 5H, Ar), 8.31 (s, 1H, –NH) ppm. Mass: *m/z* (%): 366 (M^+^, 6.31), 275 (22.72), 183 (24.94), 91 (100.0). C_21_H_22_N_2_O_4_ (366): calculated, %: C 68.84, H 6.05, N 7.65, O 17.47; found, %: C 68.82, H 6.10, N 7.55, O 17.46. Yield quartz (65%), granite (62%).

*5-Acetyl-4-(3-(benzyloxy)phenyl)-6-methyl-3,4-dihydropyrimidin-2(1H)-one* **(Ip)**. mp. = 192–194°C. I.R (KBr): *ν* = 3450, 3200, 2950, 1640, 1590 cm^−1^. ^1^H NMR (300 MHz, DMSO): *δ* = 2.08 (s, 3H, CH_3_), 2.27 (s, 3H, –COCH_3_), 5.05 (s, 2H, –O–CH_2_–Ph), 5.20 (s, 1H, –CH–), 6.81–6.92 (m, 4H, Ar), 7.21–7.45 (m, 5H, Ar), 7.81 (s, 1H, –NH), 9.17 (s, 1H, –NH) ppm. Mass: *m*/*z* (%): 336 (M^+^, 1.6), 293 (1.9), 245 (33), 153 (14.6), 91 (100.0). C_20_H_20_N_2_O_3_ (336): calculated, %: C 71.41, H 5.99, N 8.33, O 14.27; found, %: C 71.40, H 6.0, N 8.30, O 14.21. Yield quartz (40%), granite (63%).

*Ethyl-4-(2,3-dimethoxyphenyl)-6-methyl-2-oxo-1,2,3,4-tetrahydropyrimidine-5-carboxylate* **(Iq)**. mp. = 178–180°C. I.R (KBr): *ν* = 3250, 3100, 2950, 1700, 1640 cm^−1^. ^1^H NMR (300 MHz, CDCl_3_): *δ* = 1.11 (t, *J* = 7.2 Hz, 3H, –O–CH_2_–CH_3_), 2.40 (s, 3H, CH_3_), 3.87 (s, 3H, –OCH_3_), 3.92 (s, 3H, –OCH_3_), 4.06 (q, 2H, –O–CH_2_–CH_3_), 5.71 (s, 1H, –CH–), 5.72 (s, 1H, –NH), 6.73 (d, 1H, Ar), 6.87 (d, 1H, Ar), 6.98 (t, 1H, Ar), 7.27 (s, 1H, –NH) ppm. Mass: *m/z* (%): 320 (M^+^, 13.7), 288 (100.0), 243 (55.2), 183 (94.9), 155 (68.9), 137 (60.1), 77 (47.3). C_16_H_20_N_2_O_5_ (320): calculated, %: C 59.99, H 6.29, N 8.74, O 24.97; found, %: C 59.95, H 6.22, N 8.70, O 24.99. Yield quartz (63%), granite (60%).

*Ethyl-4-(3-(benzyloxy)phenyl)-6-methyl-2-thioxo-1,2,3,4-tetrahydropyrimidine-5-carboxylate* **(IVg)**. mp. = 180–182°C. I.R (KBr): *ν* = 3400, 3150, 2950, 1650 cm^−1^. ^1^H NMR (300 MHz, DMSO): *δ* = 1.10 (t, *J* = 7.2 Hz, 3H, –O–CH_2_–CH_3_), 2.27 (s, 3H, CH_3_), 4.01 (q, 2H, –O–CH_2_–CH_3_), 5.05 (s, 2H, –O–CH_2_–Ph), 5.15 (s, 1H, –CH–), 6.80 (m, 4H, Ar), 7.23–7.42 (m, 5H, Ar), 9.60 (s, 1H, –NH), 10.30 (s, 1H, –NH) ppm. Mass: *m/z* (%): 382 (M^+^, 17.6), 199 (12.2), 91 (100.0). C_21_H_22_N_2_O_3_S (382): calculated, %: C 65.95, H 5.80, N 7.32, O 12.55, S 8.38; found, %: C 65.89, H 5.60, N 7.35, O 17.46, S 8.36. Yield quartz (66%), granite (60%).

*Ethyl-4-(2,3-dimethoxyphenyl)-6-methyl-2-thioxo-1,2,3,4-tetrahydropyrimidine-5-carboxylate* **(IVc)**. mp. = 181–183°C. I.R (KBr): *ν* = 3200, 3100, 2950, 1705 cm^−1^. ^1^H NMR (300 MHz, CDCl_3_): *δ* = 1.10 (t, *J* = 7.2 Hz, 3H, –O–CH_2_–CH_3_), 2.42 (s, 3H, CH_3_), 3.86 (s, 3H, –OCH_3_), 3.93 (s, 3H, –OCH_3_), 4.05 (q, 2H, –O–CH_2_–CH_3_), 5.71 (s, 1H, –CH–), 6.68 (d, 1H, Ar), 6.88 (d, 1H, Ar), 6.99 (t, 1H, Ar), 7.26 (s, 1H, –NH), 8.10 (s, 1H, –NH) ppm. Mass: *m*/*z* (%): 336 (M^+^, 76.4), 305 (86.1), 289 (81.5), 263 (100.0), 199 (87.7), 171 (63.3), 153 (31.5), 77 (44.6). C_16_H_20_N_2_O_4_S (336): calculated, %: C 57.12, H 5.99, N 8.33, O 19.02, S 9.53; found, %: C 57.13, H 5.96, N 8.35, O 19.06, S 9.51. Yield quartz (64%), granite (63%).

*Ethyl-4-(2,5-dimethoxyphenyl)-6-methyl-2-thioxo-1,2,3,4-tetrahydropyrimidine-5-carboxylate* **(IVh)**. mp. = 188–190°C. I.R (KBr): *ν* = 3200, 3100, 2940, 1700 cm^−1^. ^1^H NMR (300 MHz, DMSO): *δ* = 1.05 (t, *J* = 8.2 Hz, 3H, –O–CH_2_–CH_3_), 2.27 (s, 3H, CH_3_), 3.65 (s, 3H, –OCH_3_), 3.72 (s, 3H, –OCH_3_), 3.96 (q, 2H, –O–CH_2_–CH_3_), 5.44 (s, 1H, –CH–), 6.57 (s, 1H, Ar), 6.83 (d, 1H, Ar), 6.94 (d, 1H, Ar), 9.24 (s, 1H, –NH), 10.24 (s, 1H, –NH) ppm. Mass: *m/z* (%): 338 (M^+^, 10.97), 279 (10.49), 256 (19.23), 166 (45.38), 149 (100.0), 105 (28.13), 69 (90.57). C_16_H_20_N_2_O_4_S (336): calculated, %: C 57.12, H 5.99, N 8.33, O 19.02, S 9.53; found, %: C 57.14, H 5.96, N 8.30, O 19.04, S 9.56. Yield quartz (64%), granite (60%).

*Ethyl-4-(2,6-dichlorophenyl)-6-methyl-2-thioxo-1,2,3,4-tetrahydropyrimidine-5-carboxylate* **(IVf)**. mp. = 222–224°C. I.R (KBr): *ν* = 3150, 3000, 2900, 1750, 1640 cm^−1^. ^1^H NMR (300 MHz, CDCl_3_): *δ* = 0.94 (t, *J* = 6.6 Hz, 3H, –O–CH_2_–CH_3_), 2.23 (s, 3H, CH_3_), 3.91 (q, 2H, –O–CH_2_–CH_3_), 6.28 (s, 1H, –CH–), 7.05–7.23 (m, 3H, Ar), 7.94 (s, 1H, –NH), 8.32 (s, 1H, –NH) ppm. Mass: *m/z* (%): 344 (M^+^, 23.9), 348 (M^+4^, 7.6), 315 (34.5), 199 (100.0), 171 (36.4), 153 (16.2). C_14_H_14_Cl_2_N_2_O_2_S (344): calculated, %: C 48.70, H 4.09, Cl 20.54, N 8.11, O 9.27, S 9.29; found, %: C 48.72, H 4.04, Cl 20.50, N 8.13, O 9.29, S 9.30. Yield quartz (55%), granite (60%).

*4-Amino-6-(3-(benzyloxy)phenyl)-5,6-dihydro-1,3,5-triazin-2(1H)-one* **(III)**. mp. = 172–174°C I.R (KBr): *ν* = 3450, 3300, 1640 cm^−1^. ^1^H NMR (300 MHz, DMSO): *δ* = 5.07 (s, 2H, –CH_2_–), 5.43 (s, 1H, –CH–), 5.68 (s, 2H, –NH_2_), 6.70–6.92 (m, 4H, Ar), 6.98 (s, 1H, NH), 7.23–7.46 (m, 5H, Ar), 10.0 (s, 1H, NH) ppm. The ^13^C-NMR (300 MHz, DMSO) *δ* = 51.2 (CH aliphatic), 62.32 (CH_2_ aliphatic), 165.3 (C triazine ring), 190.4 (C=O amidic), 111–160 (benzene rings). Mass: *m/z* (%): 296 (M^+^, 54.58), 294 (82.76), 253 (57.20), 227 (63.30), 203 (100.0), 182 (31.01), 171 (55.77), 131 (99.09), 104 (29.73). C_16_H_16_N_4_O_2_ (296): calculated, %: C 64.85, H 5.44, N 18.91, O 10.80; found, %: C 64.82, H 5.40, N 18.93, O 10.82. Yield quartz (25%).

#### 5.1.2. General Procedure of Methylation of Compounds **IVa–c**

A mixture of **IVa–c** (0.005 mol) and methyl iodide (0.005 mol) was dissolved in dry acetone in the presence of pot. Carbonate anhydrous was refluxed in water bath for 5 hours. The reaction mixture was filtered on hot then kept for overnight. The formed solid was filtered off and crystallized from an appropriate solvent to give **Va,b,c**.

*Ethyl-2-(methylthio)-4,6-diphenyl-1,6-dihydropyrimidine-5-carboxylate* **(Va)**. The formed solid was crystallized from petroleum ether/benzene 2 : 1; mp. = 158–160°C; I.R (KBr): *ν* = 3280, 2982, 2807, 1676, 1614, 1493 cm^−1^; ^1^H NMR (300 MHz, CDCl_3_): *δ* = 0.87 (t, *J* = 6.9 Hz, 3H, –O–CH_2_–CH_3_), 2.49 (s, 3H, –SCH_3_), 3.86 (q, 2H, –O–CH_2_–CH_3_), 5.74 (s, 1H, –CH–), 7.27 (s, 1H, NH), 7.28–7.48 (m, 10H, Ar) ppm; mass: *m*/*z* (%): 352 (M^+^, 23.35), 337 (33.58), 323 (68.32), 275 (100.0), 77 (25.52); C_20_H_20_N_2_O_2_S (352): calculated, %: C 68.16, H 5.72, N 7.95, O 9.08, S 9.10; Found, %: C 68.13, H 5.75, N 7.97, O 9.10, S 9.12; yield (77%).

*1-(6-(4-Methoxyphenyl)-4-methyl-2-(methylthio)-1,6-dihydropyrimidin-5-yl)ethanone* **(Vb)**. The formed solid was crystallized from petroleum ether/ethanol 1 : 1; mp. = 126–128°C; I.R (KBr): *ν* = 3285, 2950, 2928, 1639, 1594 cm^−1^; ^1^H NMR (300 MHz, DMSO): *δ* = 2.09 (s, 3H, –CH_3_), 2.23 (s, 3H, –SCH_3_), 2.28 (s, 3H, –COCH_3_), 3.70 (s, 3H, –OCH_3_), 5.54 (s, 1H, –CH–), 6.82–7.15 (d, d, 4H, Ar), 9.57 (s, 1H, –NH) ppm; mass: *m*/*z* (%): 288 (M^+^, 29.40), 250 (22.10), 183 (19.10), 73 (67.60), 57 (100.0); C_15_H_18_N_2_O_2_S (290): calculated, %: C 62.04, H 6.25, N 9.65, O 11.02, S 11.04; found, %: C 62.03, H 6.26, N 9.65, O 11.04, S 11.03; yield (86%).

*Ethyl-6-(2,3-dimethoxyphenyl)-4-methyl-2-(methylthio)-1,6-dihydropyrimidine-5-carboxylate* **(Vc)**. The solid product was crystallized from petroleum ether (60–80); mp. = 140°C; I.R (KBr): *ν* = 3321, 2935, 2832, 1706, 1669, 1594 cm^−1^; ^1^H NMR (300 MHz, DMSO): *δ* = 1.13 (t, *J* = 7.5 Hz, 3H, CH_3_CH_2_O–), 2.39 (s, 3H, –CH_3_), 2.45 (s, 3H, –SCH_3_), 3.87 (s, 3H, –OCH_3_), 3.93 (s, 3H, –OCH_3_), 4.06 (q, 2H, CH_3_CH_2_O–), 5.85 (s, 1H, –CH), 6.77–7.02 (m, 3H, Ar), 7.27 (s, 1H, –NH) ppm; mass: *m/z* (%): 350 (M^+^, 20.56), 335 (36.97), 321 (64.29), 303 (40.44), 213 (100.0), 77 (23.14); C_17_H_22_N_2_O_4_S (350): calculated, %: C 58.27, H 6.33, N 7.99, O 18.26, S 9.15; found, %: C 58.26, H 6.33, N 7.97, O 18.27, S 9.16; yield (74%).

#### 5.1.3. Acylation of Compounds **IVa–c**

*Method (A).* A mixture of **IVa**–**c** (0.005 mol) and acetyl chloride (0.01 mol) was refluxed in DMF (15 mL) as a solvent containing (5 drops) of triethylamine (TEA) for 1 hour and then stirred at room temperature for overnight, and then the solution was poured into ice with vigorous stirring, and then the solid product was filtered off and recrystallized from suitable solvent to afford compounds **VIa,b,c**.

*Method (B).* A solution of **IVa**–**c** (0.01 mol) in 15 mL of acetic anhydride was heated under reflux for 1.30 hour. The solution was then poured into 150 mL of ice-water and stirred for several hours until crystallization was complete. The precipitate was filtered and crystallized from suitable solvent to afford compounds **VIa,b,c**.

*Ethyl 3-acetyl-4-(2,3-dimethoxyphenyl)-6-methyl-2-thioxo-1,2,3,4-tetrahydropyrimidine-5-carboxylate* **(VIc)**. The solid product was recrystallized from ethanol; *method (A)*: yield (81%). *method (B)*: yield (78%); mp. = 186°C; I.R (KBr): *ν* = 3237, 2996, 2944, 2837, 1702, 1671 cm^−1^; ^1^H NMR (300 MHz, DMSO): *δ* = 1.17 (t, *J* = 6.6 Hz, 3H, CH_3_CH_2_O–), 2.27 (s, 3H, –CH_3_), 2.60 (s, 3H, –COCH_3_), 3.67 (s, 3H, –OCH_3_), 3.77 (s, 3H, –OCH_3_), 4.08 (q, 2H, CH_3_CH_2_O–), 5.50 (s, 1H, –CH), 6.68–7.0 (m, 3H, Ar), 11.6 (s, 1H, –NH) ppm; mass: *m*/*z* (%): 378 (M^+^, 48.03), 335 (96.81), 289 (100.0), 263 (45.57), 199 (33.62), 77 (22.44); C_18_H_22_N_2_O_5_S (378): calculated, %: C 57.13, H 5.86, N 7.40, O 21.14, S 8.47; found, %: C 57.14, H 5.85, N 7.41, O 21.13, S 8.45.

*Ethyl  3-acetyl-4,6-diphenyl-2-thioxo-1,2,3,4-tetrahydropyrimidine-5-carboxylate* **(VIa)**. The solid product was recrystallized from benzene; *method (A)*: yield (80%); *method (B)*: yield (85%). mp. = 136°C; I.R (KBr): *ν* = 3215, 2986, 1642, 1599, 1494 cm^−1^; mass: *m*/*z* (%): 380 (M^+^, 37.39), 337 (100.0), 307 (25.7), 265 (57.72), 104 (65.03); C_21_H_20_N_2_O_3_S (380): calculated, %: C 66.29, H 5.30, N 7.36, O 12.62, S 8.43; found, %: C 66.28, H 5.31, N 7.35, O 12.63, S 8.42.

*1,1*′*-(6-(4-Methoxyphenyl)-4-methyl-2-thioxo-2,3-dihydropyrimidine-1,5(6H)-diyl)diethanone* **(VIb)**. The solid product was recrystallized from ethanol; *method (A)*: yield (65%); *method (B)*: yield (70%). mp. = 130°C; I.R (KBr): *ν* = 3243, 2962, 1698, 1609, 1509 cm^−1^.

#### 5.1.4. Synthesis of Compounds ** (VIIa,b)**

A solution of **Va,b **(0.01 mol) in 15 mL of acetic anhydride was heated under reflux for one hour. The solution was then poured into 150 mL of ice-water and stirred for several hours until crystallization was complete. The precipitate was filtered off and washed with water then crystallized from an appropriate solvent to afford **VIIa**,**b**.

*Ethyl  1-acetyl-2-(methylthio)-4,6-diphenyl-1,6-dihydropyrimidine-5-carboxylate* **(VIIa)**. The solid product crystallized from petroleum ether (60–80); yield (90%); mp. = 102°C; I.R (KBr): *ν* = 2978, 1697, 1601, 1533 cm^−1^; ^1^H NMR (300 MHz,CDCl_3_): 0.97 (t, *J* = 6.6 Hz, 3H, CH_3_CH_2_O–), 2.50 (s, 3H, –SCH_3_), 2.50 (s, 3H, –COCH_3_), 4.02 (q, 2H, CH_3_CH_2_O–), 6.66 (s, 1H, –CH), 7.27–7.60 (m, 10H, Ar) ppm; mass: *m*/*z* (%): 394 (M^+^, 12.51), 351 (100.0), 337 (10.86), 323 (28.06), 275 (84.24), 129 (18.40), 77 (29.31); C_22_H_22_N_2_O_3_S (394): calculated, %: C 66.98, H 5.62, N 7.10, O 12.17, S 8.13; found, %: C 66.97, H 5.63, N 7.09, O 12.18, S 8.12.

*1,1*′*-(6-(4-Methoxyphenyl)-4-methyl-2-(methylthio)pyrimidine-1,5(6H)-diyl)diethanone* **(VIIb)**. The precipitated product was crystallized from benzene; yield (86%); mp. = 180°C. I.R (KBr): *ν* = 2990, 1675, 1568 cm^−1^; mass: *m*/*z* (%): 332 (M^+^, 43.22), 312 (39.56), 278 (51.28), 100 (100.0), 67 (50.55); C_17_H_20_N_2_O_3_S (332): calculated, %: C 61.42, H 6.06, N 8.43, O 14.44, S 9.65; found, %: C 61.43, H 6.04, N 8.46, O 14.45, S 9.66.

*Synthesis of Ethyl 1-Methyl-2-(methylthio)-4,6-diphenyl-1,6-dihydro-pyrimidine-5-carboxylate* **(VIIIa)**. A mixture of **Va** (0.005 mol) and methyl iodide (0.005 mol) was dissolved in DMF in the presence of pot. Carbonate anhydrous was refluxed in water bath for 4 hours. The reaction mixture was filtered on hot then the filtrate was cooled and poured onto cold water with stirring; the formed solid was filtered off and crystallized from ethanol : benzene (3 : 1); mp. = 92°C; I.R (KBr): *ν* = 3058, 2977, 2932, 1717, 1580 cm^−1^; ^1^H NMR (300 MHz, CDCl_3_): *δ* = 0.84 (t, *J* = 6.9 Hz, 3H, –O–CH_2_–CH_3_), 2.57 (s, 3H, –SCH_3_), 3.33 (s, 3H, –NCH_3_), 4.01 (q, 2H, –O–CH_2_–CH_3_), 5.74 (s, 1H, –CH–), 7.49–7.64 (m, 10H, Ar) ppm; mass: *m*/*z* (%): 366 (M^+^, 5.21), 350 (100.0), 321 (28.48), 129 (30.96), 77 (15.87); C_21_H_22_N_2_O_2_S (366): calculated, %: C 68.82, H 6.05, N 7.64, O 8.73, S 8.75; found, %: C 68.81, H 6.04, N 7.65, O 8.73, S 8.75; yield (25%). 

#### 5.1.5. General Procedure for the Preparation of Compounds **(Xa,b)**

*Method (A)*. A mixture of **IVa,b** (1 mmol) and chloroacetic acid (1 mmol) was dissolved in 40 mL of a mixture of (AC)_2_O/ACOH (1 : 3) in the presence of 3 gm anhydrous sodium acetate that was refluxed for 4 hours. The reaction mixture was cold and poured onto cold water with stirring; the solid formation was filtered off and crystallized from benzene/ethanol (3 : 1) to give **Xa,b**.

*Method (B)*. A mixture of **IVa,b** (0.005 mol) and chloroacetyl chloride with 2 drops of T.E.A was refluxed in benzene for 3 hours. Then the reaction mixture was filtered off through heating then dried and crystallized from benzene/ethanol (3 : 1) to give **Xa,b**.

*Method (C)*. A mixture of **IVa,b** (0.005 mol), ethylchloro acetate (0.005 mol), and sodium acetate trihydrate (1 gm) was refluxed in ethanol for 5 hours. The reaction mixture was filtered and kept for overnight. The solid formation was filtered off then dried and crystallized from benzene/ethanol 3/1 to afforded **Xa,b**.

*Ethyl 3-oxo-5,7-diphenyl-3,5,8,8a-tetrahydro-2H-thiazolo[3,2-a]pyrimidine-6-carboxylate* **(Xa)**. The formed solid was crystallized from benzene/ethanol (3 : 1); *method (A)*: yield (72%); *method (B)*: yield (75%); *method (C)*: yield (84%). mp. = 136–8°C; I.R (KBr): *ν* = 2976, 2931, 2900, 1752, 1675, 1589 cm^−1^; ^1^H NMR (300 MHz, CDCl_3_): *δ* = 0.85 (t, *J* = 6.9 Hz, 3H, –O–CH_2_–CH_3_), 3.85 (q, 2H, –O–CH_2_–CH_3_), 3.88 (s, 2H, –CH_2_CO–), 6.19 (s, 1H, –CH–), 7.27–7.51 (m, 10H, Ar) ppm; mass: *m*/*z* (%): 378 (M^+^, 60.03), 350 (16.30), 301 (100.0), 273 (35.62), 129 (25.11), 77 (35.57); C_21_H_18_N_2_O_3_S (378): calculated, %: C 66.65, H 4.79, N 7.40, O 12.68, S 8.47; found, %: C 66.65, H 4.78, N 7.41, O 12.67, S 8.47.

*6-Acetyl-5-(4-methoxyphenyl)-7-methyl-8,8a-dihydro-2H-thiazolo[3,2-a]pyrimidin-3(5H)-one* **(Xb)**. The formed solid was crystallized from benzene and drops of ethanol; *method (A)*: yield (42%). *method (B)*: yield (34%). *method (C)*: yield (40%). mp. = 160–162°C; I.R (KBr): *ν* = 2983, 2936, 2876, 1756, 1655, 1612 cm^−1^; ^1^H NMR (300 MHz, DMSO): *δ* = 2.16 (s, 3H, CH_3_), 2.34 (s, 3H, –COCH_3_), 3.70 (s, 3H, OCH_3_), 4.15 (s, 2H, –CH_2_CO–), 5.98 (s, 1H, –CH–), 6.86–7.21 (d, d, 4H, Ar) ppm; mass: *m*/*z* (%): 316 (M^+^, 35.08), 301 (5.32), 273 (100.0), 245 (28.08), 230 (4.72), 181 (19.36), 115 (25.14), 77 (26.0); C_16_H_18_N_2_O_3_S (316): calculated, %: C 60.36, H 5.70, N 8.80, O 15.08, S 10.07; found, %: C 60.35, H 5.70, N 8.81, O 15.06, S 10.08.

#### 5.1.6. Synthesis of Compounds **(XIa,b,c)**

A mixture of compound **Xa** (1 mmol), aromatic aldehyde (1 mmol), and 2 drops of piperidine was refluxed in ethanol for 2 hours. The reaction mixture kept overnight, then the solid product was filtered off and crystallized from EtOH to afford compound **XI**.

*(E)-Ethyl 2-(4-chlorobenzylidene)-3-oxo-5,7-diphenyl-3,5-dihydro-2H-thiazolo[3,2-a]pyrimidine-6-carboxylate* **(XIa)**. mp. = 164–6°C; I.R (KBr): *ν* = 3446, 1721, 1620, 1559 cm^−1^; ^1^H NMR (300 MHz, CDCl_3_): *δ* = 0.87 (t, *J* = 6.9 Hz, 3H, –O–CH_2_–CH_3_), 3.89 (q, 2H, –O–CH_2_–CH_3_), 6.34 (s, 1H, –CH–), 7.27–7.54 (m, 14H, Ar), 7.74 (s, 1H, =CH–) ppm; mass: *m*/*z* (%): 500 (M^+2^, 45.80), 503 (M^+4^, 9.82), 423 (67.99), 168 (100.0), 77 (77.69); C_28_H_21_ClN_2_O_3_S (500): calculated, %: C 67.13, H 4.22, Cl 7.08, N 5.59, O 9.58, S 6.40; found, %: C 67.14, H 4.24, Cl 7.07, N 5.60, O 9.59, S 6.42; yield (88%).

*(E)-Ethyl 2-(2,3-dimethoxybenzylidene)-3-oxo-5,7-diphenyl-3,5-dihydro-2H-thiazolo[3,2-a]pyrimidine-6-carboxylate* **(XIb)**. mp. = 148°C; I.R (KBr): *ν* = 34454, 1712, 1630, 1581 cm^−1^; ^1^H NMR (300 MHz, CDCl_3_): *δ* = 0.87 (t, *J* = 6.6 Hz, 3H, –O–CH_2_–CH_3_), 3.77 (s, 3H, –OCH_3_), 3.85 (s, 3H, –OCH_3_), 6.34 (s, 1H, –CH–), 6.84 (d, 1H Ar), 6.96 (s, 1H, Ar), 6.98 (d, 1H, Ar), 7.35–7.55 (m, 10H, Ar), 8.09 (s, 1H, =CH–) ppm; mass: *m*/*z* (%): 526 (M^+1^, 42.98), 449 (100.0), 363 (27.0), 77 (5.37); C_30_H_26_N_2_O_5_S (526): calculated, %: C 68.42, H 4.98, N 5.32, O 15.19, S 6.09; found, %: C 68.43, H 4.99, N 5.33, O 15.20, S 6.10; Yield (85%). 

*(E)-Ethyl2-(3-nitrobenzylidene)-3-oxo-5,7-diphenyl-3,5-dihydro-2H-thiazolo[3,2-a]pyrimidine-6-carboxylate* **(XIc)**. mp. = 158–160°C; I.R (KBr): *ν* = 3100, 2998, 1716, 1617, 1557, 1563, 1530 cm^−1^; ^1^H NMR (300 MHz, CDCl_3_): *δ* = 0.84 (t, *J* = 6.9 Hz, 3H, –O–CH_2_–CH_3_), 3.86 (q, 2H, –O–CH_2_–CH_3_), 6.31 (s, 1H, –CH–), 7.20–7.75 (m, 14H, Ar), 8.31 (s, 1H, =CH–) ppm; C_28_H_21_N_3_O_5_S (511): calculated, %: C 65.74, H 4.14, N 8.21, O 15.64, S 6.27; found, %: C 65.75, H 4.13, N 8.22, O 15.63, S 6.28; yield (92%).

*Synthesis of 1-[5-Acetyl-6-(4-methoxy-phenyl)-4-methyl-1,6-dihydro-pyrimidin-2-ylsulfanyl]-propan-2-one* **(XIII)**. A mixture of **IVb** (0.005 mol), chloroacetone (0.005 mol), and 2 drops of piperidine was refluxed in ethanol for 6 hours. The reaction mixture was kept for overnight. The solid formation was filtered off then dried and crystallized from benzene/ethanol (1 : 1); mp. = 215°C; I.R (KBr): *ν* = 3112, 3004, 1644, 1608, 1524 cm^−1^; ^1^H NMR (300 MHz, DMSO): *δ* = 2.25 (s, 3H, –CH_3_), 2.26 (s, 3H, –COCH_3_), 2.30 (s, 3H, –COCH_3_), 2.46 (s, 2H, CH_2_), 3.71 (s, 3H, –OCH_3_), 6.44 (s, 1H, –CH), 6.90–7.28 (d, d, 4H, Ar), 7.11 (s, 1H, –NH); mass: *m*/*z* (%): 332 (M^+^, 5.30), 298 (5.30), 270 (33.60), 245 (25.7), 91 (100.0); C_17_H_20_N_2_O_3_S (332): calculated, %: C 61.42, H 6.06, N 8.43, O 14.44, S 9.65; found, %: C 61.43, H 6.05, N 8.43, O 14.42, S 9.66; yield (48%).

#### 5.1.7. Reaction of Compounds **Va,b** with Thiosemicarbazide

A mixture of **Va,b** (0.005 mol) and thiosemicarbazide (0.07 mol) was refluxed in ethanol (20 mL) for 7 hours in a water bath, then the reaction mixture was allowed to stand for several hours at room temperature, then the solid product was filtered off and recrystallized from ethanol to formed compounds **XIVa,b**.

*Ethyl 2-(2-carbamothioylhydrazinyl)-4,6-diphenyl-1,6-dihydro-pyrimidine-5-carboxylate* **(XIVa)**. Method (A): yield (51%); method (B): yield (46%); mp. = 170–172°C I.R (KBr): *ν* = 3370, 3262, 3175, 1644, 1620 cm^−1^. ^1^H NMR (300 MHz, DMSO): *δ* = 0.71 (t, *J* = 7.5 Hz, 3H, OCH_2_CH_3_), 3.74 (q, 2H, OCH_2_CH_3_), 4.50 (s, 2H, –NH_2_), 5.26 (s, –CH), 7.19–7.58 (m, 10H, Ar), 8.65 (s, 1H, –NH), 9.78 (s, 1H, –NH), 10.51 (s, 1H, –NH) ppm; mass: *m*/*z* (%): 395 (M^+^, 24.13), 379 (21.13), 368 (49.20), 352 (76.97), 105 (86.51), 55 (100.0); C_20_H_21_N_5_O_2_S (395): calculated, %: C 60.74, H 5.35, N 17.71, O 8.09, S 8.11; found, %: C 60.73, H 5.36, N 17.71, O 8.08.

*2-(5-Acetyl-6-(4-methoxyphenyl)-4-methyl-1,6-dihydropyrimidin-2-yl)hydrazinecarbothioamide* **(XIVb)**. Method (A): yield (64%); method (B): yield (48%); mp. = 92°C; I.R (KBr): *ν* = 3284, 3145, 2049, 1604, 1510 cm^−1^; mass: *m*/*z* (%): 333 (M^+^, 12.18), 274 (14.92), 233 (27.31), 215 (84.93), 178 (90.61), 136 (100.0), 76 (65.0), 51 (23.07); C_15_H_19_N_5_O_2_S (333): calculated, %: C 54.04, H 5.74, N 21.01, O 9.60, S 9.62; found, %: C 54.05, H 5.74, N 21.0, O 9.61, S 9.62. 

#### 5.1.8. Synthesis of Compounds **XVIIa,b**

Compound **IVa,b** (0.005 mol) was heated under reflux in (10 mL) of methanol, containing acetic acid (2.5 mL) and water (2.5 mL). After reflux for 25 hours, methanol was distilled off and the remaining solution was treated portionwise with water until precipitation was completed. After standing for several hours at room temperature, the solid product was removed by filteration to yield **XVIIa,b** which crystallized from ethanol.

*Ethyl  2-oxo-4,6-diphenyl-1,2,3,4-tetrahydropyrimidine-5-carboxylate* **(XVIIa)**. mp. (found) = 160°C, mp. (reported) = 158°C [48]; yield (77%).

*5-Acetyl-4-(4-methoxyphenyl)-6-methyl-3,4-dihydropyrimidin-2(1H)-one* **(XVIIb)**. mp. (found) = 164–166°C; mp. (reported) = 166-167°C [48]; yield (71%). 

## Figures and Tables

**Table 1:** 3,4-Dihydropyrimidin-2(1*H*)ones (**I**).

Entry	R_1_	R_2_	Time (h)		MP. [Reference]		Yield^a^	
			Quartz	Granite	Found	Reported	Quartz	Granite
**Ia**	C_6_H_5_	OEt	3	3.5	201-202	200–202 [33]	68	64
**Ib**	2-(OH)-C_6_H_4_	OEt	3.5	5	202–204	200–202 [34]	62	60
**Ic**	4-(OCH_3_)-C_6_H_4_	OEt	3	3	199-200	201-202 [35]	64	60
**Id**	Ph-CH=CH	OEt	3	4	232–234	232–235 [36]	58	55
**Ie**	2,5-(OCH_3_)-C_6_H_3_	OEt	4	5	210–212	212–214 [37]	61	58
**If**	3,4,5-(OCH_3_)-C_6_H_2_	OEt	3	4.5	180-181	180–182 [25]	60	56
**Ig**	2-furyl	OEt	3	4	205-206	203–205 [38]	66	61
**Ih**	2-(Cl)-C_6_H_4_	OEt	3	4	214-215	215-216 [39]	65	62
**Ii**	4-(OCH_3_)-C_6_H_4_	CH_3_	3	4	165–167	166–168 [40]	69	65
**Ij**	4-N(CH_3_)_2_-C_6_H_4_	OEt	3	4	233–235	230–232 [25]	62	58
**Ik**	4-(CH_3_)-C_6_H_4_	OEt	2	3	210–212	214-215 [35]	65	62
**Il**	2,6-(Cl)-C_6_H_3_	OEt	4	4.5	302-303	305 [41]	57	61
**Im**	2-thienyl	OEt	2	3	214–216	215–217 [42]	66	63
**In**	4-(F)-C_6_H_4_	OEt	2	3	175–177	175–177 [43]	71	68
**Io**	3-(OCH_2_Ph)-C_6_H_4_	OEt	3	3.5	178–180	New	65	62
**Ip**	3-(OCH_2_Ph)-C_6_H_4_	CH_3_	3.5	4	192–194	New	40	63
**Iq**	2,3-(OCH_3_)-C_6_H_3_	OEt	3	4	178–180	New	63	60

^a^Isolated yield.

**Table 2:** 3,4-Dihydropyrimidin-2(1*H*)-thiones (**IV**).

Entry	R_1_	R_2_	R_3_	Time (h)		MP. [Reference]		Yield^a^	
				Quartz	Granite	Found	Reported	Quartz	Granite
**IVa**	C_6_H_5_	OEt	Ph	3	3	183-184	183–185 [45]	63	60
**IVb**	4-(OCH_3_)-C_6_H_4_	CH_3_	CH_3_	3.5	4	181-182	183-184 [46]	67	65
**IVc**	2,3-(OCH_3_)-C_6_H_3_	OEt	CH_3_	3.5	4	181–183	New	64	63
**IVd**	4-(OCH_3_)-C_6_H_4_	OEt	CH_3_	3	4	152-153	150–152 [43]	59	56
**IVe**	2-(OH)-C_6_H_4 _	OEt	CH_3_	3	4	210-211	206–208 [47]	63	59
**IVf**	2,6-(Cl)-C_6_H_3_	OEt	CH_3_	4	5	222–224	New	55	60
**IVg**	3-(OCH_2_Ph)-C_6_H_4_	OEt	CH_3_	3	4	180–182	New	66	60
**IVh**	2,5-(OCH_3_)-C_6_H_3_	OEt	CH_3_	4.5	5	188–190	New	64	60

^a^Isolated yield.

**Table 3:** Inhibition zone resulted from the effect of the antibiotic (Streptophenicol) and tested compounds on *Escherichia  coli* and *Staphylococcus  aureus*.

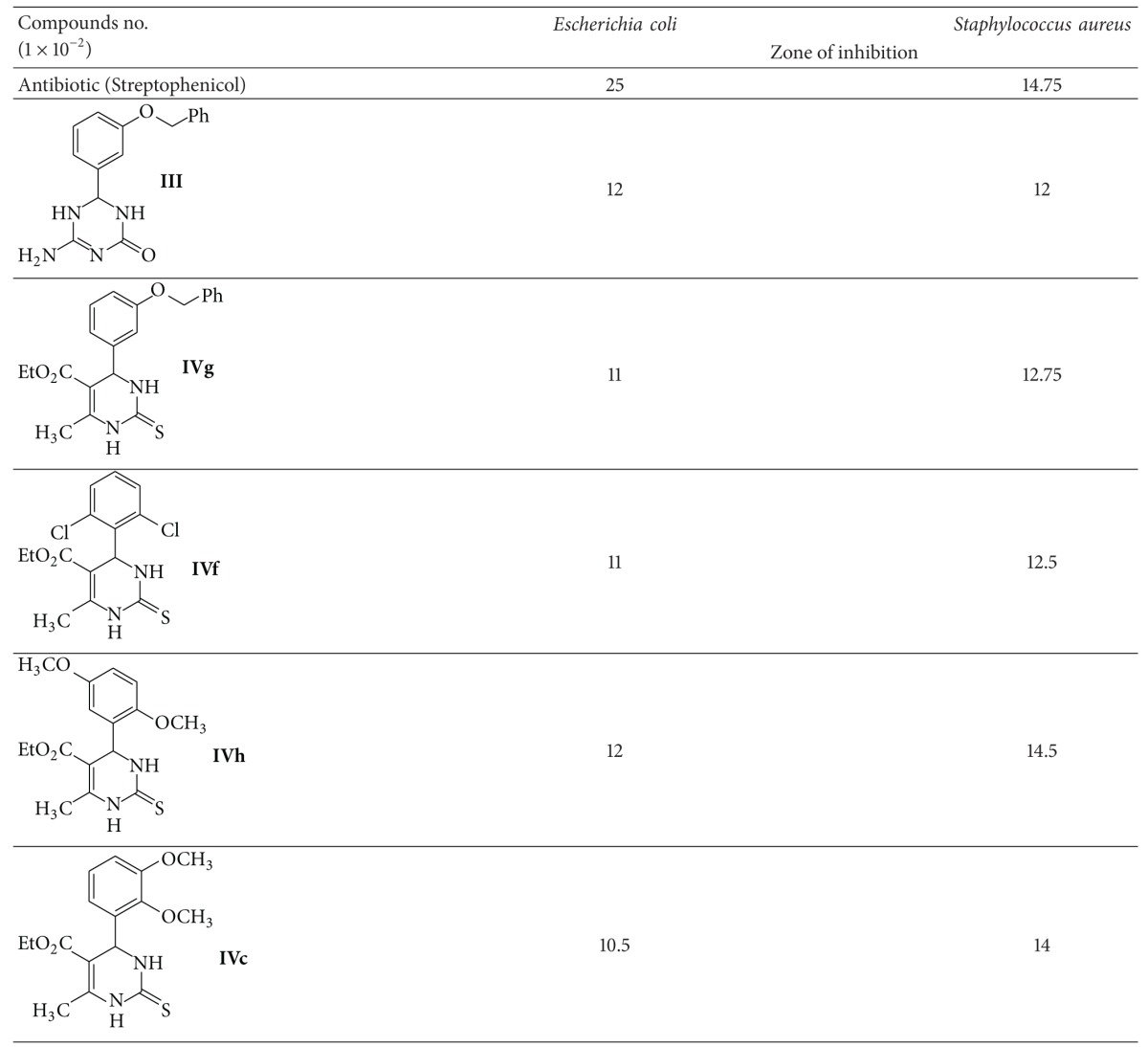

## References

[1] Hurst EW, Hull R (1961). Two new synthetic substances active against viruses of the psittacosis-lymphogranuloma- trachoma group. *Journal of Medicinal Chemistry*.

[2] Ashok M, Holla BS, Kumari NS (2007). Convenient one pot synthesis of some novel derivatives of thiazolo[2,3-*b*]dihydropyrimidinone possessing 4-methylthiophenyl moiety and evaluation of their antibacterial and antifungal activities. *European Journal of Medicinal Chemistry*.

[3] Bahekar SS, Shinde DB (2004). Synthesis and anti-inflammatory activity of some [4,6-(4-substituted aryl)-2-thioxo-1,2,3,4-tetrahydro-pyrimidin-5-yl]-acetic acid derivatives. *Bioorganic & Medicinal Chemistry Letters*.

[4] Mayer TU, Kapoor TM, Haggarty SJ, King RW, Schreiber SL, Mitchison TJ (1999). Smart molecule inhibitor of mitotic spindle bipolarity identified in a phenotype-based screen. *Science*.

[5] Kappe CO (2000). Recent advances in the Biginelli dihydropyrimidine synthesis. New tricks from an old dog. *Accounts of Chemical Research*.

[6] Patil AD, Kumar NV, Kokke WC (1995). Novel alkaloids from the sponge Batzella sp.: inhibitors of HIV gp120-human CD4 binding. *Journal of Organic Chemistry*.

[7] Snider BB, Chen J, Patil AD, Freyer AJ (1996). Synthesis of the tricyclic portions of batzelladines A, B and D. Revision of the stereochemistry of batzelladines A and D. *Tetrahedron Letters*.

[8] Biginelli P (1893). Derivati aldeidureidici degli eteri acetil-ed ossalacetico. *Gazzetta Chimica Italiana*.

[9] Wipf P, Cunningham A (1995). A solid phase protocol of the Biginelli dihydropyrimidine synthesis suitable for combinatorial chemistry. *Tetrahedron Letters*.

[10] Kappe CO (1993). 100 years of the Biginelli dihydropyrimidine synthesis. *Tetrahedron*.

[11] Dallinger D, Stadler A, Kappe CO (2004). Solid- and solution-phase synthesis of bioactive dihydropyrimidines. *Pure and Applied Chemistry*.

[12] Kappe CO (1998). 4-aryldihydropyrimidines via the Biginelli condensation: aza-analogs of nifedipine-type calcium channel modulators. *Molecules*.

[13] Kappe CO, Stadler A (2004). The Biginelli dihydropyrimidine synthesis. *Organic Reactions*.

[14] Simon C, Constantieux T, Rodriguez J (2004). Utilisation of 1,3-dicarbonyl derivatives in multicomponent reactions. *European Journal of Organic Chemistry*.

[15] Kappe CO (2003). The generation of dihydropyrimidine libraries utilizing Biginelli multicomponent chemistry. *QSAR and Combinatorial Science*.

[16] Zaugg HE, Martin WB (1965). *α*-amidoalkylations at carbon. *Organic Reactions*.

[17] Kappe CO (2005). The Biginelli reaction. *Multicomponent Reactions*.

[18] Gong LZ, Chen XH, Xu XY (2007). Asymmetric organocatalytic biginelli reactions: a new approach to quickly access optically active 3,4-dihydropyrimidin-2-(1*H*)-ones. *Chemistry—A European Journal*.

[19] Dondoni A, Massi A (2001). Parallel synthesis of dihydropyrimidinones using Yb(III)-resin and polymer-supported scavengers under solvent-free conditions. A green chemistry approach to the Biginelli reaction. *Tetrahedron Letters*.

[20] Shaabani A, Bazgir A (2004). Microwave-assisted efficient synthesis of spiro-fused heterocycles under solvent-free conditions. *Tetrahedron Letters*.

[21] Ahn BJ, Gang MS, Chae K, Oh Y, Shin J, Chalg W (2008). A microwave-assisted synthesis of 3,4-dihydro-pyrimidin-2-(1*H*)-ones catalyzed by FeCl_3_-supported Nanopore Silica under solvent-free conditions. *Journal of Industrial and Engineering Chemistry*.

[22] Zhang X, Li Y, Liu C, Wang J (2006). An efficient synthesis of 4-substituted pyrazolyl-3,4-dihydropyrimidin-2(1*H*)-(thio)ones catalyzed by Mg(ClO_4_)_2_ under ultrasound irradiation. *Journal of Molecular Catalysis A*.

[23] Li JT, Han JF, Yang JH, Li TS (2003). An efficient synthesis of 3,4-dihydropyrimidin-2-ones catalyzed by NH_2_SO_3_H under ultrasound irradiation. *Ultrasonics Sonochemistry*.

[24] Foroughifar N, Mobinikhaledi A, Fathinejad Jirandehi H (2003). Synthesis of some biginelli compounds in solvent medium using a photochemistry method. *Phosphorus, Sulfur and Silicon and the Related Elements*.

[25] Hu EH, Sidler DR, Dolling UH (1998). Unprecedented catalytic three component one-pot condensation reaction: an efficient synthesis of 5-alkoxycarbonyl-4-aryl-3,4-dihydropyrimidin- 2(1*H*)-ones. *Journal of Organic Chemistry*.

[26] Reddy CV, Mahesh M, Raju PVK, Babu TR, Reddy VVN (2002). Zirconium(IV) chloride catalyzed one-pot synthesis of 3,4-dihydropyrimidin-2(1*H*)-ones. *Tetrahedron Letters*.

[27] Kumar A, Maurya RA (2007). An efficient bakers’ yeast catalyzed synthesis of 3,4-dihydropyrimidin-2-(1*H*)-ones. *Tetrahedron Letters*.

[28] Zhang YQ, Wang C, Li GS, Li JC, Liu HM, Wu QH (2005). One-pot Synthesis of 3,4-Dihydropyrimidin-2(1*H*)-ones Catalyzed by Expandable Graphite. *Chinese Journal of Organic Chemistry*.

[29] Yarapathi RV, Kurva S, Tammishetti S (2004). Synthesis of 3,4-dihydropyrimidin-2(1*H*)ones using reusable poly(4-vinylpyridine-co-divinylbenzene)-Cu(II)complex. *Catalysis Communications*.

[30] Azizian J, Mohammadi AA, Karimi AR, Mohammadizadeh MR (2006). KAl(SO_4_)_2_
*·*12H_2_O supported on silica gel as a novel heterogeneous system catalyzed biginelli reaction: one-pot synthesis of di-hydropyrimidinones under solvent-free conditions. *Applied Catalysis A*.

[31] Mizuno N, Misono M (1998). Heterogeneous catalysis. *Chemical Reviews*.

[32] Bakibaev AA, Filimonov VD (1991). Synthesis of hydrogenated acridine-1,8-diones & 1,4-dihydropyrimidines by reaction of urea with 1,3-dicarbonyl compounds. *Russian Journal of Organic Chemistry*.

[33] Yu Y, Liu D, Liu C, Luo G (2007). One-pot synthesis of 3,4-dihydropyrimidin-2(1*H*)-ones using chloroacetic acid as catalyst. *Bioorganic & Medicinal Chemistry Letters*.

[34] Paraskar AS, Dewkar GK, Sudalai A (2003). Cu(OTf)_2_: a reusable catalyst for high-yield synthesis of 3,4-dihydropyrimidin-2(1*H*)-ones. *Tetrahedron Letters*.

[35] Gohain M, Prajapati D, Sandhub S (2004). A novel Cu-catalysed three-component one-pot synthesis of dihydropyrimidin-2(1*H*)-ones using microwaves under solvent free conditions. *Synlett*.

[36] Ma Y, Qian C, Wang L, Yang M (2000). Lanthanide triflate catalyzed Biginelli reaction. one-pot synthesis of dihydropyrimidinones under solvent-free conditions. *The Journal of Organic Chemistry*.

[37] Kamble VT, Muley DB, Atkore ST, Dakore SD (2010). Three component reaction: an efficient synthesis of 3,4-dihydropyrimidin-2(1*H*)-ones and thiones using heterogeneous catalyst. *Chinese Journal of Chemistry*.

[38] Gangadasu B, Palaniappan S, Rao VJ (2004). One-pot synthesis of dihydropyrimidinones using polyaniline-bismoclite complex. A facile and reusable catalyst for the biginelli reaction. *Synlett*.

[39] Mitra AK, Banerjee K (2003). Clay catalysed synthesis of dihydropyrimidinones under solvent-free conditions. *Synlett*.

[40] Mabry J, Ganem B (2006). Studies on the Biginelli reaction: a mild and selective route to 3,4-dihydropyrimidin-2(1*H*)-ones via enamine intermediates. *Tetrahedron Letters*.

[41] Ghorge T, Tahilramani R, Mehta DV (1975). Condensed heterocycles from 5-ethoxycarbonyl-6-methyltetrahydropyrimidin-2-ones. *Communications*.

[42] Fu N-Y, Yuan Y-F, Cao Z, Wang S-W, Wang J-T, Peppe C (2002). Indium(III) bromide-catalyzed preparation of dihydropyrimidinones: improved protocol conditions for the Biginelli reaction. *Tetrahedron*.

[43] Yadav JS, Reddy BVS, Sridhar P (2004). Green protocol for the biginelli three-component reaction: Ag 3PW_12_O_40_ as a novel, water-tolerant heteropolyacid for the synthesis of 3,4-dihydropyrimidinones. *European Journal of Organic Chemistry*.

[44] Folkers K, Johnson TB (1933). Researches on pyrimidines. CXXXVI. The mechanism of formation of tetrahydropyrimidines by the Biginelli reaction. *Journal of the American Chemical Society*.

[45] Carlos RD, Bernardi D, Kirsch G (2007). ZrCl_4_ or ZrOCl_2_ under neat conditions: optimized green alternatives for the Biginelli reaction. *Tetrahedron Letters*.

[46] Foroughifar N, Mobinikhaledi A, Jirandehi HF, Memar S (2003). Microwave-assisted synthesis of some BI- and tricyclic pyrimidine derivatives. *Phosphorus, Sulfur and Silicon and the Related Elements*.

[47] Ahmed B, Khan RA, Habibullah, Keshari M (2009). An improved synthesis of Biginelli-type compounds via phase-transfer catalysis. *Tetrahedron Letters*.

[48] Ramu E, Kotra V, Bansal N, Varala R, Adapa SR (2008). Green approach for the efficient synthesis of Biginelli compounds promoted by citric acid under solvent-free conditions. *RAS*Ā*YAN Journal of Chemistry*.

